# A Meta-Analysis and Review of Radiation Dose Escalation in Definitive Radiation Therapy between Squamous Cell Carcinoma and Adenocarcinoma of Esophageal Cancer

**DOI:** 10.3390/cancers16030658

**Published:** 2024-02-03

**Authors:** Yu Liou, Tien-Li Lan, Chin-Chun Lan

**Affiliations:** 1 School of Medicine, College of Medicine, National Yang Ming Chiao Tung University, No. 155, Sec. 2, Linong Street, Beitou District, Taipei City 112, Taiwan; 2 Department of Heavy Particles and Radiation Oncology, Taipei Veterans General Hospital, No. 201, Sec. 2, Shipai Rd., Beitou District, Taipei City 112, Taiwan; 3 Thoracic Surgery Group, Clinical Research Center, Department of Surgery, Changhua Christian Hospital, 135 Nanhsiao Street, Changhua City 500, Taiwan; 4 Department of Emergency and Critical Care Medicine, Changhua Christian Hospital, 135 Nanhsiao Street, Changhua City 500, Taiwan; 5 Post-Baccalaureate Medical School, National Chung Hsing University, 145 Xingda Rd., South District, Taichung City 402, Taiwan

**Keywords:** esophageal cancer, squamous cell carcinoma, adenocarcinoma, dose escalation

## Abstract

**Simple Summary:**

The standard treatment of choice for inoperable esophageal cancer is concurrent chemoradiation therapy. However, the treatment effect varies among patients. Currently, the optimal dose for esophageal cancer for different histologies is still under debate, and the guidelines still recommend using the same dose to treat both squamous cell carcinoma and adenocarcinoma despite their differences in radiation sensitivity. In this review article, we analyzed previous studies treating esophageal cancers with definitive radiotherapy, with or without concurrent chemotherapy. We found that squamous cell carcinoma shows an overall survival benefit with the use of radiation dose escalation, while no advantage was found for adenocarcinoma. Based on these findings, we suggest that further dose escalation can be performed for esophageal squamous cell carcinoma.

**Abstract:**

Esophageal cancer, ranked as the eighth most prevalent cancer globally, is characterized by a low survival rate and poor prognosis. Concurrent chemoradiation therapy (CCRT) is the standard therapy in the non-surgical treatment of localized carcinoma of the esophagus. Nevertheless, the radiation doses employed in CCRT remain notably lower compared to the curative definite chemoradiation therapy utilized in the management of other carcinomas. In order to increase the local control rates and enhance the treatment outcomes, several clinical trials have used high-dose radiation to analyze the effect of dose escalation. Despite the integration of technically advanced RT schemes such as intensity-modulated radiation therapy (IMRT), the results of these trials have failed to demonstrate a significant improvement in overall survival or local progression-free survival. In this review, we investigated previous clinical trials to determine the ineffectiveness of radiation dose escalation in the context of CCRT for esophageal cancer. We aim to clarify the factors contributing to the limited efficacy of escalated radiation doses in improving patient outcomes. Furthermore, we delve into recent research endeavors, exploring prospective radiation dose modifications being altered based on the histological characteristics of the carcinoma. The exploration of these recent studies not only sheds light on potential refinements to the existing treatment protocols but also seeks to identify novel approaches that may pave the way for more efficacious and personalized therapeutic strategies for esophageal cancer management.

## 1. Introduction

Esophageal cancer is one of the most aggressive cancers, and its prevalence has progressively increased. It is estimated that there will be 63.5% more new cases of esophageal cancer in 2024 than in 2020 [1]. The conventional treatments for esophageal cancer were surgery and radiotherapy (RT), with limited efficacy. The median overall survival rate was less than 10 months, and the 5-year overall survival was less than 10 percent [2]. Since the prospective randomized trial RTOG 85-01 was published in the 1990s, the standard therapy in non-surgical treatment for localized esophageal cancer has shifted to concurrent chemoradiation therapy (CCRT) instead of RT alone [2,3,4]. Owing to advanced techniques of diagnosis and treatment, the global age-standardized mortality between 1990 and 2020 has decreased by 27.5% [1].

Nevertheless, esophageal cancer is still the sixth leading cause of cancer death in the world [5]. The 5-year overall survival of patients with esophageal cancer ranges from 15% to 25% [6]. Furthermore, the recurrence risk after CCRT is considerable. The rate of locoregional recurrence after definitive chemoradiation therapy ranges from 40% to 60% [6]. From the aspect of the improvement of RT in esophageal cancer, dose escalation should be one of the considerations for more effective treatment. The current radiation dose of CCRT is a maximum of 50.4 Gy. Compared to another type of carcinoma over the chest, lung cancer, the maximum dose in stereotactic body radiation therapy (SBRT) for central tumors can be tolerated up to 70 Gy based on guidelines. In addition, radiation dose escalation can improve the outcomes in patients with non-small cell lung cancer when treated at a high dose in the range of 63–103 Gy [7]. Several clinical trials were designed to evaluate the strength of radiation dose escalation in CCRT to treat esophageal cancer, yet the results did not reveal further benefits. The phase III trial INT 0123 in 2002 used 64.8 Gy as the high radiation dose versus the standard dose of 50.4 Gy and showed no advantage in overall survival or regional control [8]. Two decades passed, and as the IMRT technique was successfully established and could precisely target the primary tumor region, the ARTDECO study performed simultaneous integrated boost (SIB) radiation therapy with up to a total dose of 61.6 Gy to the primary tumor [9]. Compared to the standard dose of 50.4 Gy, however, elevating the radiation dose to the primary tumor up to 61.6 Gy did not yield a noteworthy improvement in local progress-free survival.

Nevertheless, a limited number of studies have investigated a comparative analysis of the dose-escalating effect on squamous cell carcinoma (SCC) and adenocarcinoma (AC) of esophageal cancer. Additionally, considering the prevailing histological distribution of esophageal cancer, SCC exhibits a higher prevalence than AC [10]. Notably, a significant proportion of participants in clinical trials have been sourced from the pool of esophageal SCC cases. Theoretically, SCC is postulated to possess a heightened sensitivity to radiation, implying a potentially more favorable treatment outcome compared to AC when subjected to CCRT [11]. Consequently, the primary objective of this review article is to figure out the therapeutic implications of radiation dose escalation, specifically evaluating its impact on SCC and AC. Through a comprehensive analysis, we aim to unravel the differences between histology, specifically SCC and AC, and the therapeutic efficacy of radiation dose escalation in the management of esophageal cancer.

## 2. Material and Methods

### 2.1. Study Selection

This review article encompassed a systematic exploration of the PubMed database, focusing on meta-analyses and systematic review articles spanning the timeframe from 2002 to 2021. We concentrated on clinical trials and cohort studies predominantly addressing the treatment efficacy of definitive RT. Emphasis was placed on discerning variations in the overall survival rates across different histological types. Consequently, studies lacking clear proportions or case numbers concerning the histology types were excluded from consideration. To facilitate a more refined comparison of the overall survival rates, we exclusively incorporated studies reporting 1, 2, 3, or 5-year survival rates, excluding those reporting solely the median overall survival time or progression-free survival. After detailed selection, a total of 31 research studies were identified for subsequent in-depth analysis.

### 2.2. Endpoints of Interest

Data on the survival rates at 1, 2, 3, or 5 years, as originally presented in the source articles, were collated for analysis. Patients were stratified into four distinct groups based on the administered radiation dose, distinguishing between high-dose (>60) and standard-dose (≤60) RT, and further categorized by histology type, distinguishing between SCC and AC, as delineated by the radiation dose parameters defined in the respective studies.

### 2.3. Calculation of the Overall Survival Rate

The determination of the overall survival rates for each histological type in various years was executed using a weighted average computation, factoring in the case numbers of each study. This approach ensured that studies with larger case numbers exerted a proportionally greater influence on the overall results.

### 2.4. Limitations

It is imperative to acknowledge certain limitations inherent in this study. Primarily, China accounted for the majority of case numbers (66%) within this study, and no adjustments were made to account for population size differences among countries. Considering China’s remarkable population size relative to other nations, the absence of such adjustments introduces a potential bias. Furthermore, the inclusion of studies focusing on definitive RT means that survival rates attributable to RT alone were also considered, potentially impacting the overall survival rate. This decision was made despite past research asserting superior treatment outcomes for CCRT compared to RT alone. Additionally, the study refrained from categorizing the specific types of chemotherapy employed. Given the potential influence of diverse cytotoxic drugs on CCRT efficacy, this non-classification introduces a potential source of bias. Last but not least, AC is notably more amenable to surgical resection due to the location of the primary tumor. Consequently, the present review incorporates a notably smaller case number for AC compared to SCC.

## 3. Result

### 3.1. Study Characteristics

Within the 30 studies listed in Table 1, 6 articles specifically addressed the impact of dose escalation, incorporating findings from the pivotal INT 0123 trial. This comprehensive review yielded 18 datasets on the overall survival for high-dose (≥60) RT and 21 datasets for standard-dose (<60) RT. Notably, the study population spanned a spectrum, with the largest cohort comprising 3977 cases and the smallest encompassing 18 cases. Further delineating the histological composition, SCC accounted for 8616 cases, while AC constituted 807 cases.

Geographically, the distribution of participating institutes exhibited a predominant prevalence in China (27%) and Japan (27%), followed by the United States (23%) and Europe (17%). This diverse representation ensures a comprehensive examination of regional variations in treatment practices and outcomes.

In addition to survival data, attention was directed toward the assessment of adverse effects utilizing the Common Terminology Criteria for Adverse Events (CTCAE) 4.0 grading system. Specifically, the focus extended to adverse events over grade 3, with a particular emphasis on the gastrointestinal and hematological systems. Gastrointestinal complications, prominently manifested as esophagitis and dysphagia, bore a direct correlation with the primary target of RT. Hematological adversities, primarily leucopenia or neutropenia, suggested myelotoxicity stemming from either RT or CCRT. These nuanced insights into adverse events contribute crucial information to the holistic evaluation of the therapeutic landscape.

We categorized the studies based on several key parameters, including the case numbers for squamous cell carcinoma (SCC) and adenocarcinoma (AC), the composition of the study population, the median dose with the accompanying dose range, the annual overall survival rates, and the evaluation of adverse effects (AE) utilizing the Common Terminology Criteria for Adverse Events (CTCAE) 4.0 grading system. The cumulative proportion of adverse events was documented, with a specific focus on prevalent gastrointestinal (GI) and hematologic (Hema) adverse effects observed in each study. 

### 3.2. Survival Rate Trends in SCC and AC

**Figure 1:**
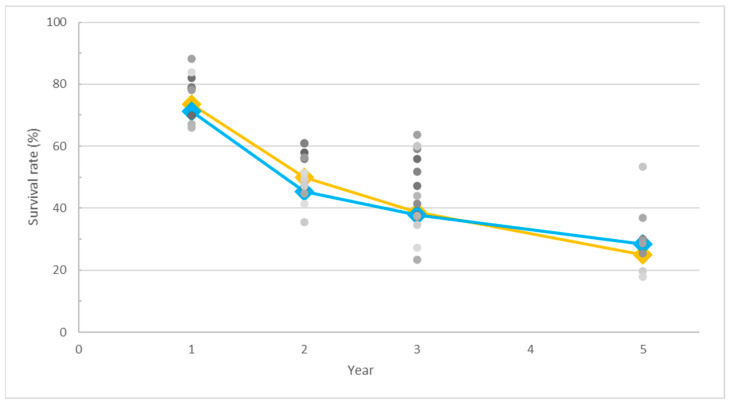
The gray dots show the actual survival rates for both squamous cell carcinoma (SCC) and adenocarcinoma (AC). Different gray dots represent each particular study. We considered the number of cases to analyze the survival trends and created the line on the graph. The blue line represents SCC, and the light orange line represents AC in the overall survival curve.

As depicted in Figure 1, the raw data points, represented by gray dots, elucidate the overall survival rates for both SCC and AC. Employing a weighted average approach based on case numbers, the survival rate trends were generated, resulting in the depiction of an overall survival curve. The OS curve portrays SCC with a blue line and AC with a light orange line.

The initial and second-year raw data points exhibited a range hovering around 35%. This observation raises considerations regarding the potential impact of the limitations outlined in the methodology, introducing a source of bias that may influence the interpretation of the results. Furthermore, the divergence in the survival rates among studies widened notably in the middle to late periods of our analytical focus, ranging from 40% to 50%. This increased variability underscores the influence of diverse therapeutic strategies during the specified timeframe of survival assessment. It is noteworthy that the fifth year saw a reduction in case numbers, potentially limiting the robustness of our analysis for that specific timeframe. The inclusion of additional data on long-term survival rates would be invaluable in refining the accuracy of the trend analysis, particularly concerning the extended prognosis beyond the fifth year. Such supplementary data could contribute to a more comprehensive and nuanced understanding of the prolonged trajectory of survival outcomes in our study cohort.

The temporal dynamics of the survival rate trends for both histological groups exhibited noteworthy similarities. In the initial year, comparable survival rates were observed for both SCC and AC. However, a notable decline ensued from the first to the second year, indicative of a critical period post-treatment. Subsequently, the rate of decline decelerated, leading to a more gradual incline in survival rates from the second to the fifth year.

Notably, the OS curve for AC surpassed that of SCC, until a divergence in trends was observed in the fifth year. This indicated the difference in long-term survival outcomes between the two histological subtypes, suggesting the extended survival trends for SCC may be more favorable than those for AC. These observations provide valuable insights into the temporal dynamics of the survival rates for SCC and AC, shedding light on potential distinctions in their long-term prognoses.

### 3.3. Impact of Radiation Dose on Overall Survival in SCC and AC

**Figure 2:**
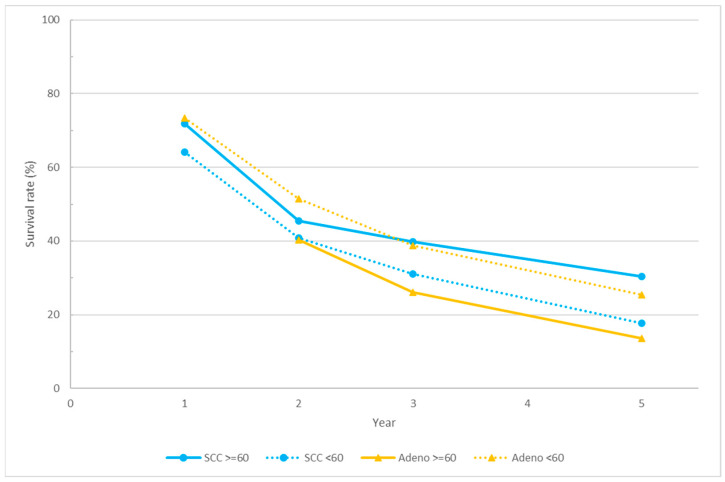
This figure illustrates the relationship between radiation dose and overall survival, categorized by histology. The blue and light orange lines show the overall survival for squamous cell carcinoma (SCC) and adenocarcinoma (AC), respectively. Solid lines represent high radiation doses while dotted lines represent standard radiation doses.

The investigation delved into the nuanced dynamics of the overall survival rates between squamous cell carcinoma (SCC) and adenocarcinoma (AC) under varying radiation dose regimens. Figure 2 provides a graphical representation of the interplay between radiation dose and overall survival, stratified by histology. The blue and light orange lines represent the overall survival of SCC and AC, respectively, with solid lines and dotted lines indicating high and standard radiation doses.

Within the SCC treatment, the high-dose group demonstrated a superior overall survival rate compared to the standard-dose group in the initial year, persisting with this advantage until the fifth year. Both groups experienced a decline from the first to the second year; however, the high-dose group exhibited a more gradual decline in the second to the fifth year, indicative of a more favorable outcome. Conversely, in AC treatment, while the overall survival rate in the first year for the high-dose group was not available, the trends suggested a diminished survival rate associated with high-dose radiation, with the standard-dose group consistently exhibiting a superior survival rate.

An in-depth analysis based on histological subtypes revealed that SCC in the high-dose group presented a more favorable overall survival than AC in the high-dose group, implying heightened radiosensitivity in SCC. Interestingly, despite the similar survival rate in the second year, SCC in the standard-dose group demonstrated superior survival rates from the second to the fifth year compared to AC in the high-dose group, particularly during the critical drop from the second to the third year. Notably, AC in the standard-dose group exhibited the highest overall survival rate, akin to SCC in the high-dose group, surpassing other groups with a survival rate exceeding 50% in the second year. However, a divergence in the high-dose SCC group emerged in the third year. The trajectory of AC in both the standard- and high-dose groups mirrored that of SCC in the standard-dose group after the third year, while SCC in the high-dose group maintained a more gradual slope from the second year. Remarkably, SCC in the high-dose group sustained an overall survival rate surpassing 30% even in the fifth year, indicating the superior long-term survival of this subgroup. In contrast, AC in the high-dose group consistently exhibited the poorest survival rates from both short-term and long-term perspectives, falling below 30% in the third year and dipping below 20% in the fifth year.

The insights gleaned from Figure 2 provide a rationale for the higher overall survival rate observed in AC than in SCC, as depicted in Figure 1. Notably, the majority of studies adhered to the standard radiation dose recommended by the current guidelines, potentially contributing to the observed higher survival rates in AC under standard doses. The scarcity of studies explicitly addressing histological differences and adjusting therapy doses accordingly further underscores the need for tailored treatment approaches based on histology types, potentially elucidating the observed survival differentials.

## 4. Discussion

Esophageal cancer is considered one of the most devastating malignancies, with many patients being diagnosed at an advanced local or metastatic stage. According to previous studies, less than 30% of patients are deemed suitable for upfront surgery at the time of diagnosis [41]. For those who are not surgical candidates but still present with localized disease, the current guidelines recommend definitive concurrent chemoradiotherapy (CCRT) or pre-operative CCRT as the primary treatment approach. In the context of pre-operative CCRT, the standard radiation dose is 41.4 Gy, as established by the CROSS trial [42,43]. An intriguing observation from this phase 3 randomized study is that as adenocarcinoma constitutes three-quarters of the entire cohort, with squamous cell carcinoma being the main histology in the remaining patients, the response to radiation treatment varies significantly between these two cohorts. In the adenocarcinoma group, the addition of neoadjuvant CCRT increased the median survival from 27.1 months to 43.2 months. Surprisingly, for the squamous cell carcinoma group, the benefit of adding neoadjuvant CCRT resulted in an increase in the median survival from 21.1 months to 81.6 months. Additionally, the pathological response rates for adenocarcinoma and squamous cell carcinoma after pre-operative CCRT were 23% and 49%, respectively. The evidence presented by the CROSS trial indicates that different cancer histologies exhibit markedly distinct clinical responses to radiation. Therefore, in the setting of definitive radiation therapy, higher doses should be designed to counteract potential under-dosage for more radio-resistant cell types.

Dose escalation for the same primary cancer with different pathological types, aside from sarcomas, presents challenges in clinical radiation therapy. Currently, identical total doses and fraction sizes are commonly employed for adenocarcinoma and squamous cell carcinoma in various organs [43]. For instance, in lung cancers, the treatment design is primarily based on the tumor’s location (peripheral or central). Similarly, head and neck cancers, despite variations in their metastatic rates based on histology, often receive the same dose for local control. Even in cases such as cervical cancer, where squamous cell carcinoma frequently exhibits notable tumor shrinkage during treatment compared to less response in adenocarcinoma, the standard dose for radiation therapy remains uniform. Several hypotheses attempt to explain this phenomenon. Firstly, despite advancements in modern radiotherapy, establishing a definitive relationship between genetics and radio-sensitivity remains elusive. While numerous genes have been shown to enhance radio-sensitivity, whether by reducing the effectiveness of DNA damage repair or enhancing the production of oxidative stress, proving these effects in human studies is exceedingly challenging due to the lack of widespread personalized genetic assays [44]. The second reason is the constraint imposed by normal organs close to the primary target. Many treatment designs focus on increasing the radiation dose to the tumor, but if adjacent organs can tolerate only a certain level of radiation damage, further potential for dose escalation to the tumor becomes limited. This is because the damage to normal organs might outweigh the benefit of increased tumor control, potentially leading to enhanced local control but decreased overall survival or quality of life. The third reason is the heterogeneity among individual patients and even within different tumor cells in the same patient, making the relationship between endpoints and specific factors more complex to verify. Given these challenges, a careful and well-designed prospective dose escalation study is necessary to validate a new dose over the standard treatment.

Previous studies on dose escalation for esophageal cancer have yielded unsatisfactory results. Early investigations, such as RTOG 8501 [2], demonstrated an overall survival benefit from concurrent chemotherapy rather than dose escalation, with a significant divergence in 5-year overall survival rates (26% compared to 0%). This disparity was primarily attributed to differences in both local failure and distant metastasis. The subsequent INT 0123 study further confirmed that, with the same concurrent chemotherapy regimen, dose escalation failed to demonstrate overall survival or local control benefits [8]. Critics often point out that these trials were conducted in the late 90s when the 2D irradiation technique was predominant. Dose escalation from limited fields in this context significantly increased the toxicity in nearby organs. In the current era of radiation therapy, most institutes have upgraded their linear accelerators and treatment planning systems, utilizing intensity-modulated radiation therapy (IMRT) and volumetric-modulated arc therapy (VMAT) as the primary treatment techniques. The ARTDECO phase 3 randomized study, focusing on inoperable esophageal cancer, employed the simultaneous integrated boost (SIB) technique, comparing 50.4 Gy in 28 fractions to the primary tumor and adjacent lymphatics with a boost to the primary tumor to 61.6 Gy [9]. Both study arms received concurrent carboplatin and paclitaxel. This study did not show a significant improvement in locoregional progression-free survival due to by dose escalation in both squamous cell carcinoma and adenocarcinoma, and there was no overall survival benefit. However, the 3-year locoregional progression-free survival was 52% for the standard arm and 59% for the SIB arm (*p* = 0.08), suggesting a trend toward better local control with dose escalation. This result implies that a slightly higher boost dose may lead to a remarkable improvement in locoregional control. Another phase 3 randomized trial, CONCORDE, demonstrated no benefit in locoregional progression-free survival when comparing 50 Gy in 25 fractions and 66 Gy in 33 fractions to the tumor, with both groups delivering 40 Gy to the elective nodal regions [45]. The median locoregional progression-free survival was 16.2 months and 18.4 months, respectively (*p* = 0.88). Interestingly, the high-dose group even showed an increased toxicity associated with dose escalation.

Despite the current evidence failing to demonstrate the benefit of dose escalation, it is essential to note that most of these randomized trials did not separately analyze the outcomes based on different histology types. In the context of modern medicine, where precision medicine and personalized treatment have gained importance, comparing different treatment options based on specific disease conditions becomes crucial. The CROSS trial, evaluating the benefits of pre-operative concurrent chemoradiotherapy (CCRT), not only highlighted the advantages of adding intensive treatment before surgery but also revealed that 49% of squamous cell carcinoma patients achieved a pathological complete response compared to only 23% in adenocarcinomas. In our review, we aggregated data from previous studies that provided the overall survival outcomes for squamous cell carcinoma and adenocarcinoma treated with definitive radiotherapy, with or without concurrent chemotherapy. The initial analysis revealed that squamous cell carcinoma exhibited a slightly lower survival during the first 3 years compared to adenocarcinoma, with the trend reversing after the fourth year. By the fifth year, the overall survival for squamous cell carcinoma became 5% higher than adenocarcinoma. This suggests that adenocarcinomas may respond more favorably to initial treatment, although the difference between the two histologies was not significant. During the first 3 years post-treatment, the survival curves for both histologies were nearly parallel, indicating a similar disease progression pattern. To gain further insights into the long-term survival outcomes, we stratified each group based on the dose prescribed to the gross lesions for more detailed information.

The second plot compares patients with different histology types treated with 60 Gy or more and those treated with less than 60 Gy. For adenocarcinomas, the trend mirrored previous studies, indicating that higher doses resulted in inferior overall survival outcomes compared to lower doses. This pattern persisted from the early years post-treatment to long-term follow-up. In contrast, squamous cell carcinoma patients demonstrated a higher overall survival with higher doses, maintaining the trend from 1-year post-treatment to 5 years, with an increasing divergence in survival over time. Comparing all four curves, squamous cell carcinoma treated with a high dose exhibited the best overall survival, followed by adenocarcinoma with a low dose, squamous cell carcinoma with a low dose, and the worst overall survival observed in adenocarcinoma treated with a high dose. This finding suggests an essential treatment decision point, indicating that squamous cell carcinomas are radiation-sensitive and achieving better survival with dose escalation is a promising approach. As for cervical esophageal cancers, where doses up to 66 Gy for the gross tumor are frequently used, it might be beneficial to apply the same dose to tumors located in the thoracic esophagus. Regarding the worsened survival observed with dose escalation in adenocarcinomas, considering adenocarcinomas’ tendency to be diagnosed in the lower esophagus, it is plausible that the decreased survival with a higher dose is associated with an increased cardiac dose. Presently, the dose constraint for the heart, based on RTOG 0623, includes a mean dose less than 26 Gy, V100 < 40 Gy, V67 < 45 Gy, and V33 < 60 Gy. Achieving this clinical goal might be challenging given the need to limit the dose to the lung while irradiating a significant volume between both lung lobes to prevent radiation pneumonitis [46]. Limiting the dose to the spinal cord is also essential to avoid neuropathy. Considering the various risk organs adjacent to the primary site, it is inevitable to have a dose delivered to the heart. Some radiation-related cardiac toxicities, such as pericardial effusion leading to cardiac tamponade, a potentially fatal cardiac event, and pulmonary hypertension associated with radiation to the lung and the heart, pose significant risks [47,48]. Given that many esophageal cancer patients have habits such as smoking, which contribute to pulmonary issues, their baseline pulmonary condition may not be satisfactory, exacerbating the progression of pulmonary hypertension. Severe pulmonary hypertension may lead to right ventricle hypertrophy and heart failure and cause lethal damage to the patient. Additionally, radiation to the atrium and frequently overlooked cardiac conduction nodes may result in atrial fibrillation or other arrhythmic conditions [49]. While multiple studies on lung and breast irradiation have underscored the importance of lowering the dose to these cardiac structures, it is recommended that future studies planning dose escalation for esophageal cancer exercise greater caution in minimizing the radiation dose to the heart to mitigate late complications.

Particle irradiation therapies, such as proton therapy and carbon ion therapy, have gained global attention recently. The physical characteristics of the Bragg peak enable the deposition of radiation to be focused on the target volume, significantly reducing the dose to the proximal organs compared to conventional photon therapy [50,51]. Moreover, there is often no detectable dose behind the main tumor. Beyond the beam direction, the lateral dose fall-off is considerably lower than that of photon beams, providing the ability to further decrease the dose distribution to nearby organs. Another evolving technique is boron neutron capture therapy, a binary treatment involving the selective injection of boron-containing drugs into the tumor [52]. Upon irradiation by a neutron beam, the boron undergoes a fission reaction, breaking into an alpha particle and a lithium nucleus. This results in energy deposition smaller than the size of a tumor cell, sparing adjacent organs from radiation damage [53]. These treatment modalities offer advantages in treating tumors surrounded by critical organs and are expanding their treatment indications and popularity. It is foreseeable that in the near future, esophageal cancer may be indicated for particle therapies.

## 5. Conclusions

Despite the advancements in radiation techniques over the past decades, the treatment outcomes of definitive radiotherapy for esophageal cancers remain challenging. The primary obstacle lies in the difficulty of escalating the irradiation dose due to potential complications in the normal organs. In this article, we demonstrated that dose escalation to more than 60 Gy has a survival benefit for esophageal squamous cell carcinoma, while no advantage was observed for adenocarcinoma. Based on our findings, we anticipate that future studies will explore increasing doses specifically for squamous cell carcinomas. Furthermore, with the latest radiation techniques, there is potential for adenocarcinoma patients to also benefit from escalated doses to the target while minimizing damage to the nearby organs. This approach may contribute to prolonging the survival of patients with adenocarcinoma.

## Figures and Tables

**Table 1:** Study characteristics.

Author	SCC Number	AC Number	Study Population	Median Dose (Gy) (Range)	Overall Survival (y: Year)	AE ≥ 3: Total (%) (GI %, Hematology %)
Shirai K, 2011 [12]	0	20	Japan	59 (N/A)	2 y 41%	N/A
Li C, 2021 [13]	3977	0	China	60 (40–76)	1 y 69.8% 2 y 46.6% 3 y 37.9% 5 y 30.1%	N/A
Roeder F, 2014 [14]	22	3	German	56 (19.2–62)	1 y 82% 2 y 61% 3 y 56%	52 (Dysphagia 19, Leukopenia 28)
Ge X, 2015 [15]	112	0	China	N/A (60–66)	1 y 78.57% 3 y 47.32%	N/A (Esophagitis 25, Leukopenia 32.14%)
La T, 2010 [16]	6	12	USA	50.4 (34.2–58.8)	1 y 79% 2 y 56%	40 (Esophagitis/Dysphagia 46, Hema 7)
Tu L, 2013 [17]	36	0	China	60 (52–70)	1 y 83.3% 2 y 42.8%	N/A (GI 11.1, Neutropenia 13.9)
Gerber N, 2014 [18]	0	41	USA	N/A (50.4–56)	2 y 61%	N/A (Esophagitis 12.2, Hema 17.07)
Xu Y, 2016 [19]	69	0	China	N/A (50.4–56)	1 y 73.8% 2 y 57.4% 3 y 41.0%	N/A (Esophagitis 14.5, Leukopenia 14.6)
Lin S, 2012 [20]	7	25	USA (Caucasians 95.2%)	50.4 (N/A)	3 y 51.7%	N/A (Esophagitis 9.7%, N/A)
Takada A, 2016 [21]	46	1	Japan	73.4 (64.6–80)	3 y 59.2%	N/A (Esophagitis 10.6%, Leukopenia 55.3)
Freilich J, 2015 [22]	48	184	USA	N/A (40–60)	3 y 41.53%	29.7 (N/A)
He L, 2016 [23]	53	317	USA (white 84.7%)	50.4 (N/A)	3 y 37.04% 5 y 25.49%	N/A (N/A)
Yang H, 2017 [24]	78	0	China	N/A (60–70)	2 y 56.2%	15.6 (Dysphagia 12.8, hema 10.24)
Iwase H, 2013 [25]	116	0	Japan	60 (N/A)	1 y 78.2% 5 y 29.8%	N/A (Nausea 3.4, Neutropenia 37.9)
Kato K, 2013 [26]	50	1	Japan	50.4 (N/A)	1 y 88.2% 3 y 63.8%	N/A (Esophagitis 35, Leukopenia 82.3)
Nishimura Y, 2012 [27]	90	1	Japan	60 (N/A)	2 y 45% 5 y 28.57%	16.48 (Esophagitis 2.1, N/A)
Kato K, 2011 [28]	76	0	Japan	60 (N/A)	3 y 44.7% 5 y 36.8%	N/A (Esophagitis 17, Leukocytosis 43)
Conroy T, 2014 [29]	229	37	France	50 (N/A)	3 y 23.41%	N/A (Dysphagia 26.64, Neutropenia 28.96)
Conroy T, 2010 [30]	80	17	France	50 (N/A)	1 y 67.18% 3 y 37.64%	73.68 (Dysphagia 17.89, Hema 73.68)
Higuchi K, 2014 [31]	42	0	Japan	61.2 (12), 50.4 (30)	1 y 66.1% 3 y 43.9%	N/A (Esophagitis 28.6, Leukopenia 71.4)
Wang H, 2007 [32]	8	16	USA	50.4 (N/A)	3 y 60%	N/A (Nausea 19, Neutropenia 4)
Meng X, 2013 [33]	55	0	China	59.4 (N/A)	1 y 92.55% 2 y 75.20%	N/A (Mucositis 12.7, Neutropenia 32.7)
Crosby T, 2013 [34]	188	65	UK	50 (N/A)	2 y 48.65%	N/A (GI 43.5, Hema 24.5)
Ishida Y, 2019 [35]	21	0	Japan	60	3 y 60% 5 y 53.4%	N/A (Esophagitis 14.29, Neutropenia 28.57)
Minsky B, 2002 [8]	187	31	USA (white 69%, black 24%)	64.8 vs. 50.4	2 y 31% vs. 40%	76% vs. 71% (N/A)
Clavier J, 2011 [36]	113	30	France	66 vs. 50	2 y 44.7% vs. 50.8% 3 y 36.8% vs. 31.6% 5 y 19.1% vs. 20.7%	8.43 vs. 6.67 (N/A)
Suh Y, 2014 [37]	117	9	Republic of Korea	>60 (60–75.6) vs. <60 (45–59.4)	2 y 52.4% vs. 45.2%	N/A (10.4% vs. 8.2%, N/A)
Chen C, 2016 [38]	648	0	China	>60 vs. 50.4	5 y 22% vs. 14%	N/A
Deng Y, 2017 [39]	139	0	China	>59.4 vs. 50.4	1 y 89% vs. 78% 2 y 61.0% vs. 39.0% 3 y 30% vs. 24%	N/A (10.5% vs. 2.2%, N/A)
Chang C, 2017 [40]	2061	0	Taiwan	>60 (60–72) vs. <60 (45–59.4)	2 y 35.47% vs. 26.74%	>2: 22.56% (23.52% vs. 21.78%)
